# Semi-quantitative measurement of specific proteins in human cumulus cells using reverse phase protein array

**DOI:** 10.1186/1477-7827-11-100

**Published:** 2013-10-22

**Authors:** Vincent Puard, Thibaud Tranchant, Veronique Cadoret, Christophe Gauthier, Eric Reiter, Fabrice Guerif, Dominique Royere

**Affiliations:** 1 UMR85 Physiologie de la Reproduction et des Comportements, INRA, F-37380 Nouzilly, France; 2 UMR7247 Physiologie de la Reproduction et des Comportements, CNRS, F-37380 Nouzilly, France; 3 Université François Rabelais de Tours, F-37041 Tours, France; 4 IFCE, F-37380 Nouzilly, France; 5 Médecine et Biologie de la Reproduction, CHRU Tours - Hôpital Bretonneau, Cedex 1, F-37000 Tours, France

**Keywords:** Biomarkers, Cumulus cells, Oocyte developmental competence, Reverse phase protein array

## Abstract

**Background:**

The ability to predict the developmental and implantation ability of embryos remains a major goal in human assisted-reproductive technology (ART) and most ART laboratories use morphological criteria to evaluate the oocyte competence despite the poor predictive value of this analysis. Transcriptomic and proteomic approaches on somatic cells surrounding the oocyte (granulosa cells, cumulus cells [CCs]) have been proposed for the identification of biomarkers of oocyte competence. We propose to use a Reverse Phase Protein Array (RPPA) approach to investigate new potential biomarkers of oocyte competence in human CCs at the protein level, an approach that is already used in cancer research to identify biomarkers in clinical diagnostics.

**Methods:**

Antibodies targeting proteins of interest were validated for their utilisation in RPPA by measuring siRNA-mediated knockdown efficiency in HEK293 cells in parallel with Western blotting (WB) and RPPA from the same lysates. The proteins of interests were measured by RPPA across 13 individual human CCs from four patients undergoing intracytoplasmic sperm injection procedure.

**Results:**

The knockdown efficiency of VCL, RGS2 and SRC were measured in HEK293 cells by WB and by RPPA and were acceptable for VCL and SRC proteins. The antibodies targeting these proteins were used for their detection in human CCs by RPPA. The detection of protein VCL, SRC and ERK2 (by using an antibody already validated for RPPA) was then carried out on individual CCs and signals were detected for each individual sample. After normalisation by VCL, we showed that the level of expression of ERK2 was almost the same across the 13 individual CCs while the level of expression of SRC was different between the 13 individual CCs of the four patients and between the CCs from one individual patient.

**Conclusions:**

The exquisite sensitivity of RPPA allowed detection of specific proteins in individual CCs. Although the validation of antibodies for RPPA is labour intensive, RRPA is a sensitive and quantitative technique allowing the detection of specific proteins from very small quantities of biological samples. RPPA may be of great interest in clinical diagnostics to predict the oocyte competence prior to transfer of the embryo using robust protein biomarkers expressed by CCs.

## Background

Defining the developmental ability of an embryo during in vitro fertilisation remains a major challenge both in humans and domestic mammals. Morphological criteria are most frequently used to evaluate the developmental and implantation ability of the embryos in human assisted-reproductive technology (ART). However, such morphological criteria (zygote scoring, early cleavage and embryo morphology at day 2 or 3) remain poorly predictive of developmental or implantation ability [1]. Direct studies, such as genomic or proteomic analyses, are difficult in human embryos, since such approaches remains invasive and might alter the embryo integrity [2]. Therefore ART laboratories need some indirect and non-invasive selection criteria [3].

Various studies reported on molecules inside the follicle or the embryo microenvironment through proteomic and metabolomic analysis of oocytes or embryos [4] and have identified potential biomarkers of oocyte or embryo quality. Other studies focused on the somatic cells (cumulus cells [CCs] and/or granulosa cells [GCs]) surrounding the oocyte since their interactions are involved in the acquisition of oocyte meiotic and developmental competence [5]. Indeed, specific oocyte factors are involved in the differentiation and expansion of CCs, and prevent the apoptosis and luteinisation of the oocyte-cumulus complex (see [6] for review). Via these interactions, the oocyte may promote specific patterns of gene and protein expression in CCs [7,8]. Recently, specific transcriptomes of cumulus cells were identified according to oocyte chromatin morphology in animals [9] or to oocyte chromosome status in humans [10]. Moreover, potential biomarkers of oocyte competence in humans and animals were proposed, based on expression of specific genes in CCs [11,12]. Indeed, the fact that protein translation is a highly regulated process implies that mRNA abundance does not always correlate with the level of the corresponding proteins. Such discrepancies might be explained by differences in mRNA stability, degradation/synthesis rates or post-translational modification of the proteins. Therefore, one can guess that, at least in some instances, parallel measurements of a given transcript and the corresponding protein could lead to increased robustness and predictive value of biomarkers. Despite the fact that the ultimate effectors in cells remain the proteins, few studies have addressed CC proteome analysis, mainly because of a lack of sensitivity of the techniques. The global protein expression pattern of human individual CCs was investigated by two-dimensional polyacrylamide gel electrophoresis according to ovarian stimulation protocol without identification of specific proteins linked to oocyte competence [13]. The CC proteome was explored by mass spectrometry and Western blotting (WB) on pooled CCs and specific proteins implicated in fatty acid metabolism and pre-mRNA splicing according to maternal age were highlighted [14]. However, to date no study investigating individual CC proteomes and specific proteins according to oocyte competence has been reported.

Over the last decade, protein microarray-based methods, such as Reverse Phase protein arrays (RPPA), which is a sensitive and quantitative technique allowing the detection of specific proteins in small quantities of biological samples, were applied to proteome analysis and allowed investigation of multiples target of a signalling pathway in the same biological sample [15]. Briefly, the principle of this approach is to coat biological samples from the cohort of patients on nitrocellulose-covered glass slides and probe the slides with specific antibodies. The sample is printed in serial dilutions that allow quantification. Interestingly, RPPA is a cost- and material-effective technique compared to standard approaches for biomarker validation such as ELISA or Luminex assays and has been used in cancer research to develop better markers for diagnosis, prognosis and treatment-outcome prediction [16,17]. Importantly, in the present study we used an improved RPPA procedure by combining near infrared detection and signal amplification protocols that were previously described by Dupuy et al. [18]. This approach leads to minimal background signal and an exquisite signal/noise ratio.

In the present study, we tested the ability of RPPA to detect and analyse the expression level of specific proteins in individual human cumulus cells as potential biomarkers of oocyte developmental competence. Our previous study identified a transcriptome of 308 genes in the human cumulus cells that were differentially expressed according to the oocyte developmental competence [19]. The proteins v-src avian sarcoma (Schmidt-Ruppin A-2) viral oncogene homolog (SRC) and regulator of G-protein signalling 2 (RGS2) were selected among these 308 genes. The protein vinculin (VCL) was selected as the reference protein in cumulus cells. The antibodies targeting the selected biomarkers were then validated for RPPA through small interfering RNA (siRNA)-mediated selective knockdowns in HEK293 cells. Following their validation, three antibodies were tested on individual CCs.

## Methods

### Cumulus cells recovery

**Figure 1:**
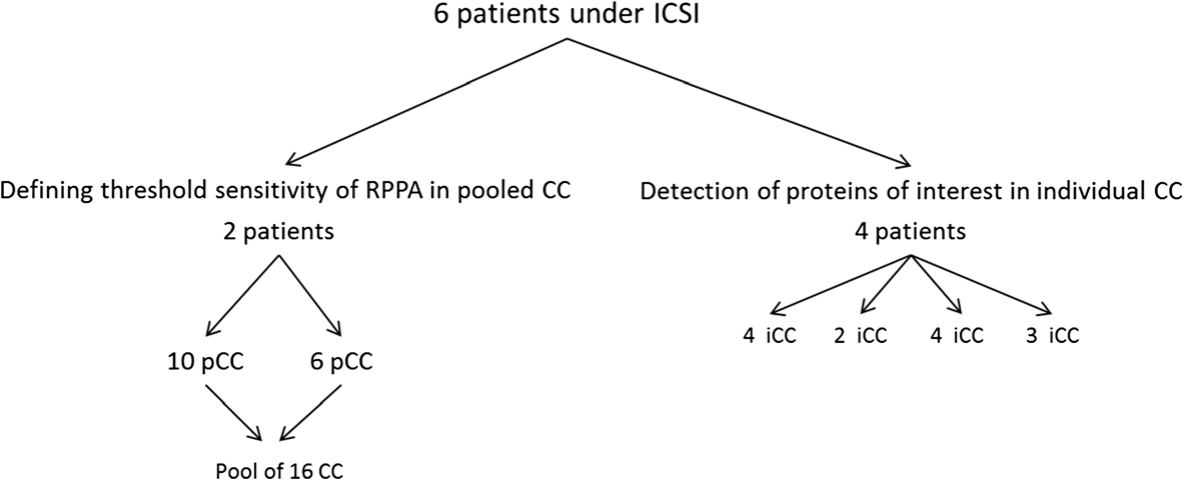
**Distribution of patients included in study.** Cumulus cells (CC) of two patients were included for defining the threshold of sensitivity of RPPA. After individual recovery, the CC of individual patients were pooled (pCC) before being pooled together. Thirteen individual CCs (iCCs) of four patients were used to test the ability of RPPA to detect specific proteins of interest.

Human cumulus cells (CCs) were collected from six patients undergoing an intracytoplasmic sperm injection (ICSI) procedure for male infertility without taking into account the oocyte or embryo competence (Figure 1). Shortly before ICSI, human CCs were individually subjected to dissociation, as already described by Feuerstein *et al.*[20]. Cumulus cells were separated from the oocyte with strippers after brief exposure to hyaluronidase (80 UI/ml, SynVitro Hyadase®, Medicult, Jyllinge, Denmark) at 37°C and were recovered individually or pooled together. Cumulus cells were washed in cold phosphate-buffered saline (PBS). CCs from pooled cumulus were centrifuged at 300 g for 5 minutes whereas the individual cumulus were dissociated then equally distributed in two tubes before the centrifugation. The supernatants were removed and the pellets were stored at -80°C until RPPA assay. Only one tube corresponding to each individual CC was used for RPPA assay, which represents 0.5 equivalent CCs, the other was stored for future analysis. The ovarian stimulation protocol, the ICSI and the embryo culture procedures have been described previously [21]. In this preliminary study, oocyte and embryo quality was not taken into account. Cumulus cells included in this study were recovered from immature oocytes and from oocytes leading to fertilisation failures.

### Cell culture and small interfering RNA (siRNA) transfection

HEK293 cells were grown in Minimum Essential Medium eagle (MEM) (Sigma-Aldrich, St Louis, MO, USA), supplemented with 10% Foetal Bovine Serum (FBS, Sigma-Aldrich) and antibiotics (100 U/mL penicillin, 100 mg/mL streptomycin) (Sigma-Aldrich) at 37°C with 5% CO_2_ in a humidified atmosphere. The siRNA transfection was done according to the manufacturer’s recommendations. Briefly, cells were seeded at a density of 2x10^4^ cells/well in 6-well plates and were then cultivated for 24 h at 37°C and 5% CO_2_. Prior to transfection, complete medium was replaced with antibiotic-free medium. Cells were transfected with 10 μl of siRNA targeting VCL, RGS2, SRC or glyceraldehyde-3-phosphate dehydrogenase (GAPDH, used as positive control), all purchased from Dharmacon (Lafayette, CO, USA). For each target, four individual siRNAs were pooled. Non-silencing siRNA control was also purchased from Dharmacon. siRNA were transfected by using 10 μl of Dharmafect (Dharmacon, Lafayette, CO, USA). Cells were harvested after 72 h of incubation.

### Cells lysis

The lysis buffer (MPER, Thermo Scientific) was supplemented with protease inhibitor (miniComplete, Roche) and phosphatase inhibitor (complete ultra tablet, Roche).

HEK293 cells transfected with siRNA were trypsinized, pelleted, suspended in 50 μl of lysis buffer and lysed for 20 min at 4°C on a rotating wheel. After centrifugation, approximately 50 μl of protein extract was obtained.

Individual and pooled CCs were suspended in 40 μl of lysis buffer and lysed for 20 min at 4°C on a rotating wheel. After centrifugation, approximately 40 μl of total protein extract was obtained.

### Western blotting (WB)

An equal volume of 2× Laëmmli buffer (32 mM Tris pH 6.8, 8% SDS, 5% beta-mercaptoethanol, 10% glycerol, 0.02% bromophenol blue) was added to HEK293 cell lysates, heated for 10 min at 95°C, resolved by 10% SDS-PAGE and transferred to nitrocellulose membrane (Protrans, Whatman, Maidstone, UK). Membranes were saturated with TBST-BSA (Tris 10 mM, pH 8, 150 mM NaCl with 0.1% Tween 20 and 4% BSA) and probed with primary antibodies (see Table 1) in TBST-BSA overnight at 4°C. Membranes were washed three times with TBST (Tris 10 mM, pH 8, 150 mM NaCl with 0.1% Tween 20) before being probed with secondary antibody (peroxidase-conjugated F(ab’)2 fragment donkey anti-rabbit IgG (1:2000) (Jackson ImmunoResearch Laboratories) or peroxidase-conjugated F(ab’)2 fragment goat anti-mouse IgG (1:2000) (Jackson ImmunoResearch Laboratories). WB was revealed with Supersignal West Pico Chemiluminescent Substrate (Pierce Biotechnology, Rockford, IL, USA). Films (Amersham Hyperfilm ECL) were scanned and the optical density of the signals was measured with ImageJ software (National Institutes of Health, USA).

**Table 1:** List of antibody targeting proteins of interest used for RPPA and WB

**Antibody name**	**Company & reference**	**Species**	**Validated for RPPA**	**Dilution RPPA**	**Dilution WB**
*ERK2 (C-14)*	Santa Cruz Biotechnology - #sc-154	Rabbit - pAb	Yes	1:1000	1:10000
*GAPDH (D16H11)*	Cell Signaling - #5174	Rabbit - mAb	No	1:1000	1:10000
*SRC (36D10)*	Cell Signaling - #2109	Rabbit - mAb	No	1:500	1:1000
*VCL*	Sigma-Aldricht - #V9131	Mouse - mAb	No	1:1000	1:10000
*RGS2*	Abnova - #H00005997-M01	Mouse - mAb	No	1:500	1:5000

### Reverse phase protein array (RPPA)

For each transfection, a five-fold dilution of cell lysate was spotted in two replicates on the array. Total cell lysate of 16 individual CCs from two patients were pooled and used to determine the threshold of sensitivity of RPPA. Samples were spotted in two replicates using a 32-pin manual arrayer (Glass Slide Microarrayer, VP478, V&P Scientific, San Diego, CA, USA). According to the manufacturer’s indications, 3–13 nL of sample was spotted on the slide (Fast Slides, Whatman, Maidstone, UK) per array pin touch. An eight-fold dilution corresponding to a range of 16 to 0.125 equivalents CCs was spotted on the array.

Total cells lysate of 13 individual CCs of four patients were analysed by RPPA and a two-fold dilution corresponding to a range of 0.5 to 0.25 equivalents CCs was spotted in two replicates.

Antibodies targeting selected proteins were the same as those used for WB. None of them were validated for RPPA prior to this study except anti-ERK2 [18]. Antibody concentrations are listed in Table 1. RPPA was adapted from [18]. Briefly, desiccated nitrocellulose-coated Fast-slides were printed with samples, using a 32-pin manual arrayer and desiccated again overnight. The immunodetection procedure was adapted from [22]. All antibodies were pre-cleared in FBS for 1 h at 37°C prior to use. After rehydration with PBS-Tween-20 0.1% (PBST), slides were blocked overnight at 4°C with 3% casein in PBST. Slides were probed with antibodies (listed in Table 1) for 3 h at room temperature (RT) in PBST with 20% FBS (PBST-FBS). Slides then were washed three times for 5 min with three rinses of PBST between washes and then probed for 45 min at RT with peroxidase-conjugated F(ab’)2 fragment donkey anti-rabbit IgG (1:2000) (Jackson ImmunoResearch Laboratories) or peroxidase-conjugated F(ab’)2 fragment goat anti-mouse IgG (1:2000) (Jackson ImmunoResearch Laboratories) diluted in PBST-FBS. Slides were washed as previously described for signal amplification and slides were subsequently incubated for 10 min at RT with BioRad Amplification Reagent (Amplified Opti-4CN Substrate Kit, BioRad, Hercules, CA, USA). Then, slides were rinsed six times, washed once for 5 min in PBST with 20% DMSO (PBST-DMSO) and washed twice for 5 min and rinsed in PBST as above. Slides were then probed for 1 h at RT with streptavidin AlexaFluor 680 conjugate (0.2 mg/mL) (AF680-streptavidin; Invitrogen LifeTechnologies) in PBST-FBS. Finally, slides were air-dried by centrifugation at 2500 rpm for 15 min and scanned at 700 nm with the Odyssey IR imaging system (LI-COR Biosciences, Lincoln, NE, USA) at 42-μm resolution.

Scanned images of arrays were analysed with GenePix Pro6.0 software (Molecular Devices, Sunnyvale, CA, USA).

### Data analysis

#### Validation of antibodies

For WB, signal intensities were determined for each point with subtraction of minimum profile background. Signal intensity of the protein of interest was normalised by the signal of VCL, which was used as a protein of reference, or by the signal of ERK2 (for the validation of anti-VCL) to calculate the relative expression level. Different exposure times were compared for each WB experiment in order to avoid signal saturation. For RPPA, the mean value of intensity was determined for each spot with subtraction of the local background intensity. The mean value of intensity of the protein of interest was normalised by the mean value of intensity of VCL (protein of reference) or by the signal of ERK2 (for the validation of anti-VCL) to calculate the relative expression level. Pearson correlation analysis was performed between the dilution and the signal intensity (GraphPad Prism 5 Software, San Diego, CA, USA). Knockdown efficiency of the targeted proteins was estimated by comparing the signal intensity of the targeted protein normalised to a reference protein (VCL and ERK2) in siRNA-treated cells versus control siRNA-treated cells (arbitrarily chosen as 100%).

#### Reverse phase protein array on human cumulus cells

The intensity of each spot was obtained after subtracting the local background intensity. Signal intensity is expressed for each sample as mean ± standard deviation (SD) of the intensity of the two replicates. The mean value of intensity of ERK2 and SRC was normalised by the mean value of intensity of VCL (protein reference) to calculate the level of expression in each individual CC.

## Results

### Validation of antibodies

**Figure 2:**
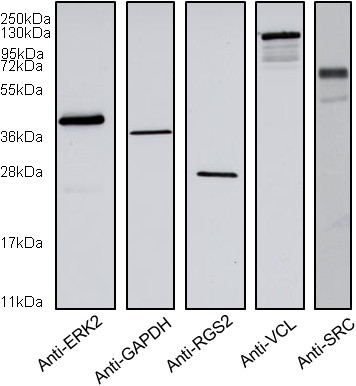
**Specificity of antibodies.** HEK293 cells lysates were analysed by Western blotting to test the specificity of antibodies.

Before their use in RPPA, the antibodies targeting the proteins of interest were assessed for their specificity by WB on HEK293 cell lysates. A major band corresponding to the expected molecular weight was detected in each lane (Figure 2). Minor bands also appeared for the antibodies targeting SRC and VCL.

**Figure 3:**
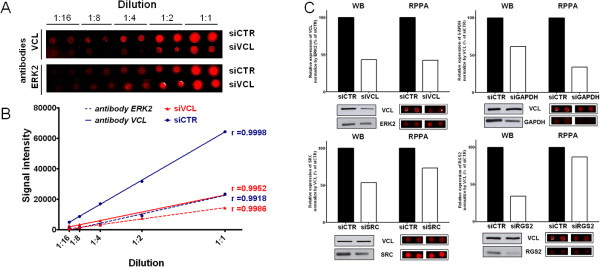
**Knockdown efficiencies of target proteins in HEK293 cells measured by Reverse Phase Protein Array (RPPA) and Western blotting (WB). A**. Detection by RPPA of VCL and ERK2 in serial dilution of HEK293 cells transfected with siRNA targeting VCL (siVCL) or non-silencing siRNA control (siCTR). **B**. Linear regression was performed for ERK2 and VCL protein and Pearson correlation between dilution and the signal intensity was calculated for each condition of Figure 3A, with all p-values lower than 0.001. Signal intensities are expressed as mean of the replicates. **C**. Level of expression by WB and RPPA of targeted proteins in HEK293 cells transfected with siRNA targeting proteins of interest or non-silencing siRNA control (siCTR). Specific bands for WB and spots corresponding to the dilution of the dynamic range in RPPA are presented.

We further assessed the antibodies’ suitability for RPPA by measuring siRNA-mediated knockdown efficiency in HEK293 cells in parallel with WB and RPPA from the same lysates. Pearson correlations were calculated for each antibody in all dilutions of HEK293 cells transfected by siRNA targeting the four proteins (VCL, SRC, RGS2 and GAPDH). Pearson correlation coefficients were above 0.98 in all conditions with all p-values lower than 0.003 (see Additional file 1: Table S1). The knockdown efficiency quantified by RPPA was calculated with the dilution 1:2 and 1:4 corresponding to an unsaturated signal (Figure 3A and Figure 3B). Using Western blotting, the knockdown efficiency for GAPDH, used as a positive control of the transfection, was estimated at 39%. The silencing of transcription was confirmed by the knockdown efficiencies of VCL and SRC (56.5% and 46% respectively) of the control. By RPPA, the knockdown efficiency for GAPDH, VCL and SRC was lower than 25% (67%, 57% and 27% respectively). The extinction level of GAPDH, VCL and SRC were acceptable in WB and in RPPA with differences in knockdown efficiencies measured by WB and RPPA for SRC and GAPDH (Figure 3C), which validated these antibodies for RPPA. However, the knockdown efficiency of RGS2 measured in RPPA was too low (13% compared to 66% measured by WB) to allow RGS2 antibody validation.

Therefore, the antibodies targeting VCL, ERK2 and SRC were used for the detection of the proteins in human CCs.

### Reverse phase protein array on human cumulus cells

Defining sensitivity and threshold of RPPA on a pool of 16 human cumulus cells.

The sensitivity of RPPA was evaluated on a pool of 16 human cumulus by assessing the signal observed with the validated antibodies using serial dilutions of the pool while the lowest level of detection was determined.

**Figure 4:**
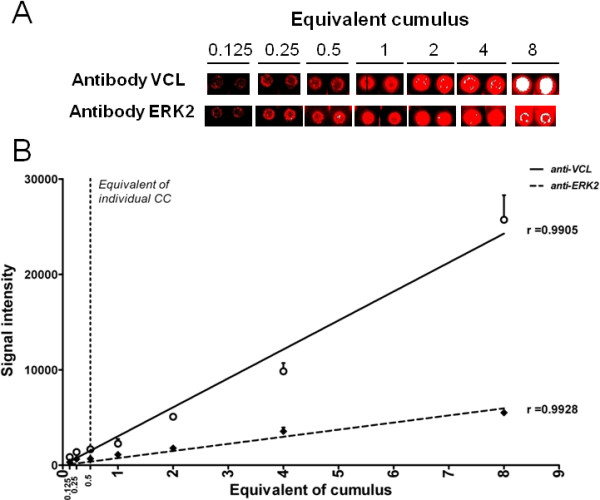
**Detection of VCL and ERK2 protein by Reverse Phase Protein Array on a pool of 16 human cumulus cells. A**. An eight-fold dilution of a pool of 16 human cumulus cells was used for the detection of VCL and ERK2 protein by Reverse Phase Protein Array. **B**. Signal intensities are expressed as mean of the two replicates. Linear regression was performed for VCL (solid line) and ERK2 (dotted line) and Pearson correlation was calculated (p < 0.0001 in both cases).

Signal intensity was saturated with all antibodies at the highest concentration (equivalent of 16 CCs spotted). Signal intensities were clearly correlated with the dilution of cells from the pool of the 16 cumulus with r = 0.99 (p < 0.0001) for both VCL and ERK2 (Figure 4), r = 0.96 (p < 0.001) for SRC and r = 0.92 (p < 0.03) for GAPDH (see Additional file 2: Table S2). Additionally, a signal was detected for the dilution corresponding to 0.25 equivalent cumulus spotted for all antibodies. After taking into account the volume printed per spot and estimating the number of cells composing a cumulus (see Additional file 3), the equivalent of 0.5 to 2 cells were printed per spot. Signals corresponding to the three antibodies were above the detection threshold.

#### Detection of proteins of interest in individual human cumulus cells

The detection of protein SRC, ERK2 and VCL was then carried out on 13 individual CCs. Signals were detected for each sample of individual CCs (*i.e.* 0.5 equivalents of individual CCs) for the three proteins and remained detectable at 0.25 equivalents of individual CCs in all 13 cumulus analysed (see Additional file 4: Figure S1). Moreover, the signal intensities of the three proteins were significantly correlated with the two dilutions corresponding to the equivalent of 0.5 CCs and 0.25 equivalents of CCs (p < 0.0001 for ERK2 and VCL, p < 0.002 for SRC respectively) (see Additional file 5: Table S3).

**Figure 5:**
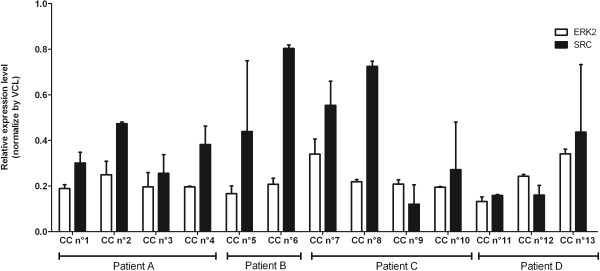
**Level of expression of ERK2 and SRC by Reverse Phase Protein Array on 13 individual cumulus cells from 4 patients.** The level of expression of ERK2 (white box) and SRC (black box) were measured by Reverse Phase Protein Array on 13 individual cumulus cells from four patients **(A to D)**. The equivalent of 0.5 individual cumulus cells were spotted in two replicates on array. Signal intensities were expressed as mean ± SD of the normalised signal of the replicates.

The level of expression of ERK2 was almost the same across the 13 individual CCs while the level of expression of SRC was different between the 13 individual CCs of the four patients and between the CCs from individual patients (Figure 5).

## Discussion

In this study, we demonstrated that RPPA combining near-infrared (NIR) dyes can be applied to human cumulus cells to measure potential biomarkers of oocyte competence. ERK2 proteins were detectable in individual human cumulus. Moreover, after validation by the siRNA approach in HEK293 cells of the antibodies targeting VCL and SRC, we were able to detect these two proteins of interest with the same sensitivity.

Defining embryo quality with non-invasive methods continues to be the major goal in ART while morphological criteria of early embryo development to predict further development or implantation remain poorly predictive [23]. Various approaches involving transcriptomics, proteomics and metabolomics on the embryo or its cellular or non-cellular environment have been proposed (see [24] for review). The cumulus cells surrounding the oocyte may be targeted to develop a non-invasive method to investigate the oocyte developmental competence and several studies have already identified biomarkers by transcriptomic analysis [11]. While the proteins remain the final effectors in the cells, no link between the product of these mRNAs (*i.e.* proteins) and the oocyte competence has been established to date. This is due, in large part, to a lack of technology sensitive enough to analyse specific proteins from the minute amount of cumulus cells available.

Protein microarray-based methods, particularly RPPA, are very sensitive and allow the detection of multiple specific targeted proteins in small quantities of biological material compared to Western blotting. Indeed, WB needs protein from 5 × 10^5^ cells whereas RPPA requires nanoliters of protein lysate (picograms to femtograms of protein) from the equivalent of 200 cells [17]. Compared to mass spectrometry, which may need further identification of the highlighted proteins, RPPA targets only specific proteins. Moreover, mass spectrometry requires heavy and expensive equipment compared to RPPA. RPPA has been successfully applied to biomarker profiling in cancer diseases (see [25] for review). In this context, we thought that RPPA could be used to evaluate the level of protein expression of specific proteins as biomarkers of oocyte developmental competence in cumulus cells. In order to achieve a proof of concept, we used two potential biomarkers RGS2 [19,26] and SRC (data not shown) and a housekeeping protein VCL, which was used as a reference protein.

As a first step, we used siRNA targeting the proteins of interest in HEK293 cells as a mean to validate the antibodies for their use in RPPA [27]. The knockdown efficiencies estimated by RPPA were close to those observed by Western blotting for SRC, VCL and GAPDH, thus allowed us to validate these antibodies for RPPA. In contrast, the knockdown efficiencies measured by WB and by RPPA for RGS2 did not allow us to validate this antibody for RPPA, even though no dominant non-specific band was observed for this antibody by WB. This might be explained by the experimental changes between the techniques. Indeed, the interactions between the epitope of the proteins and the paratope of the antibody might be modulated by the physico-chemical conditions such as the temperature, the pH of the buffers or the concentration of the detergent. Furthermore, the protein conformation might modify these interactions. Finally, the antibody targeting RGS2 was designed against a fragment of recombinant RGS2, which might lead to non-specific binding.

RPPA sensitivity was assessed on pooled then individual human CCs using the validated antibodies. The study by Dupuy et al*.*[18] showed that RPPA combined with amplified NIR dyes allow the detection of phosphorylated proteins in less than 1 ng of total cell extract. Moreover, using NIR dyes allowed them to detect ERK2 protein in the equivalent of two HEK293 cells printed per spot. This work showed the extreme sensitivity of the techniques that could be applied to the research on a small amount of biological sample such as cumulus cells. In line with this work, we showed that ERK2 protein can be detected for a dilution corresponding to less than one cell of an individual human cumulus printed per spot. Furthermore, VCL and SRC proteins were detected with the same range of sensitivity.

Even though antibody validation remains a challenging problem, RPPA seems to be a useful technology for biomarker detection for clinical diagnostics in individual cumulus cells (sensitive, high throughput, and cost- and material-effective) compared to WB, 2D electrophoresis and mass spectrometry, which have already been tested on human cumulus cells. The siRNA approach has shown its efficiency for antibody validation [27,28]. However, this approach is limited by the protein characteristics (*i.e.* turn-over of synthesis, half-life and cellular function) and requires specific development for each target. In this context, siRNA is not optimal for rapid and high throughput validation of a large collection of antibodies. Alternatively, overexpression of the targeted protein by plasmid transfection might be a better alternative for future antibody validations.

## Conclusions

In conclusion, we demonstrate for the first time that RPPA combined with NIR dyes could be applied to individual human CCs to specifically detect and quantify proteins. We validated three antibodies for RPPA use through the siRNA approach to analyse specific proteins. Thereby, RPPA could be used to target specific proteins as potential biomarkers of oocyte competence in human CCs such as ERK2 and SRC. Our next steps will be to test these two proteins in individual CCs as biomarkers of oocyte developmental competence, to validate new antibody targeting RGS2 and to search for other biomarkers at protein level from transcriptomics study.

## Competing interests

The authors declare that they have no competing interests.

## Authors’ contributions

Study design: VP, TT, ER, DR. Execution: VP, TT, CG, VC. Analysis: VP. Manuscript drafting and critical discussion: VP, ER, FG, DR. All authors read and approved the final manuscript.

## Supplementary Material

Additional file 1: Table S1 Pearson correlation analysis between the dilution of HEK293 cells lysate transfected with siRNA and the signal intensity observed by Reverse Phase Protein Array with antibodies targeting proteins of interest. Pearson correlations were calculated between the five-fold dilution of HEK293 cells lysate transfected with siRNA targeting VCL, RGS2, SRC or GAPDH (used as positive control) and the signal intensity observed by Reverse Phase Protein Array with antibodies targeting VCL, RGS2, ERK2, GAPDH or SRC. Click here for file

Additional file 2: Table S2 Pearson correlation analysis between the dilution of a pool of 16 cumulus cells and signal intensity observed by Reverse Phase Protein Array with antibodies targeting proteins of interest. Pearson correlations were calculated between the seven-fold dilutions of a pool of 16 human cumulus cells and the signal intensity observed by Reverse Phase Protein Array with antibodies targeting VCL, ERK2, GAPDH or SRC. Click here for file

Additional file 3 **Estimation of the number of cells of an individual cumulus printed per spot on the array.** The number of cells composing a cumulus was estimated to be more than 17 individual cumulus cells with a Tomas chamber. According to the manufacturer’s indications, a volume of 3–13 nL of sample is printed per spot on the array. The estimation of cells of an individual cumulus per spot was estimated with the dilutions of the pool of 16 individual cumulus cells used for Reverse Phase Protein Array. Click here for file

Additional file 4: Figure S1 Detection of ERK2, SRC and VCL proteins by Reverse Phase Protein Array on 13 individual cumulus cells. Detection of ERK2 (A), SRC (B) and VCL (C), by Reverse Phase Protein Array on 13 individual cumulus cells from four patients (A to D). The equivalent of 0.5 (white box) and 0.25 (black box) individual cumulus cells were spotted in two replicates on the array. Signal intensities are expressed as mean ± SD of the two replicates. Click here for file

Additional file 5: Table S3 Correlation between signals intensities observed by Reverse Phase Protein Array and the dilutions of the 13 individual cumulus cells. Pearson correlation were calculated between the signal intensities observed by Reverse Phase Protein Array with the antibodies targeting ERK2, SRC and VCL and the dilutions at equivalents of 0.5 and 0.25 cumulus cells for the 13 individual cumulus cells. Click here for file
