# Supplementary material for: Semi-quantitative measurement of specific proteins in human cumulus cells using reverse phase protein array
Source: Reprod Biol Endocrinol. 2013 Oct 22;11:100. doi: 10.1186/1477-7827-11-100 (PMC4015149; doi:10.1186/1477-7827-11-100)
Supplement: Additional file 3 — Estimation of the number of cells of an individual cumulus printed per spot on the array. The number of cells composing a cumulus was estimated to be more than 17 individual cumulus cells with a Tomas chamber. According to the manufacturer’s indications, a volume of 3–13 nL of sample is printed per spot on the array. The estimation of cells of an individual cumulus per spot was estimated with the dilutions of the pool of 16 individual cumulus cells used for Reverse Phase Protein Array. [file 1477-7827-11-100-S3.pdf]

Materials and Methods

Estimation of number of cells composing a cumulus

The number of cells composing a cumulus was estimated over 17 individual cumulus collected as previously described. Each cumulus was recovered in 75µl of PBS, briefly mixed to dissociate and homogenize the solution. Two counts were done with Thoma chamber and mean of the two counts were used to estimate the number of cells of a cumulus.

Results

Estimation of number of cells composing a cumulus

The average number of cells was calculated on 17 individual cumulus and represent a mean of 29500 cells per cumulus (range 11000–50750 cells). An equivalent of 0.5 CC corresponding to each individual CC used for RPPA assay represent an amount of 14500 cells in average.

Table. Conversion table between equivalent of cumulus cells, volume per spot on array and the number of equivalent cells of cumulus spotted.

**Pooled CC (from 16 individual CCs composed of a mean of 29 000 cells)**				
lysis buffer (nL)	*number of cells from 16 cumulus*	*number of equivalent cells of cumulus for 1nL*	*number of equivalent cells of cumulus for 3nL*	*number of equivalent cells of cumulus for 13nL*
40 000	464 000	12	35	151
	equivalent of individual CCs	*number of equivalent cells of cumulus for 3nL spotted*	*number of equivalent cells of cumulus for 13nL spotted*	
	16	35	151	
	8	17	75	
	4	9	38	
	2	4	19	
	1	2	9	
	0,5	***1***	***5***	
	0,25	0,5	2	
	0,125	0,3	1	
	0,0625	0,1	0,6	
