# Supplementary figures and images for: Semi-quantitative measurement of specific proteins in human cumulus cells using reverse phase protein array
Source: Reprod Biol Endocrinol. 2013 Oct 22;11:100. doi: 10.1186/1477-7827-11-100 (PMC4015149; doi:10.1186/1477-7827-11-100)

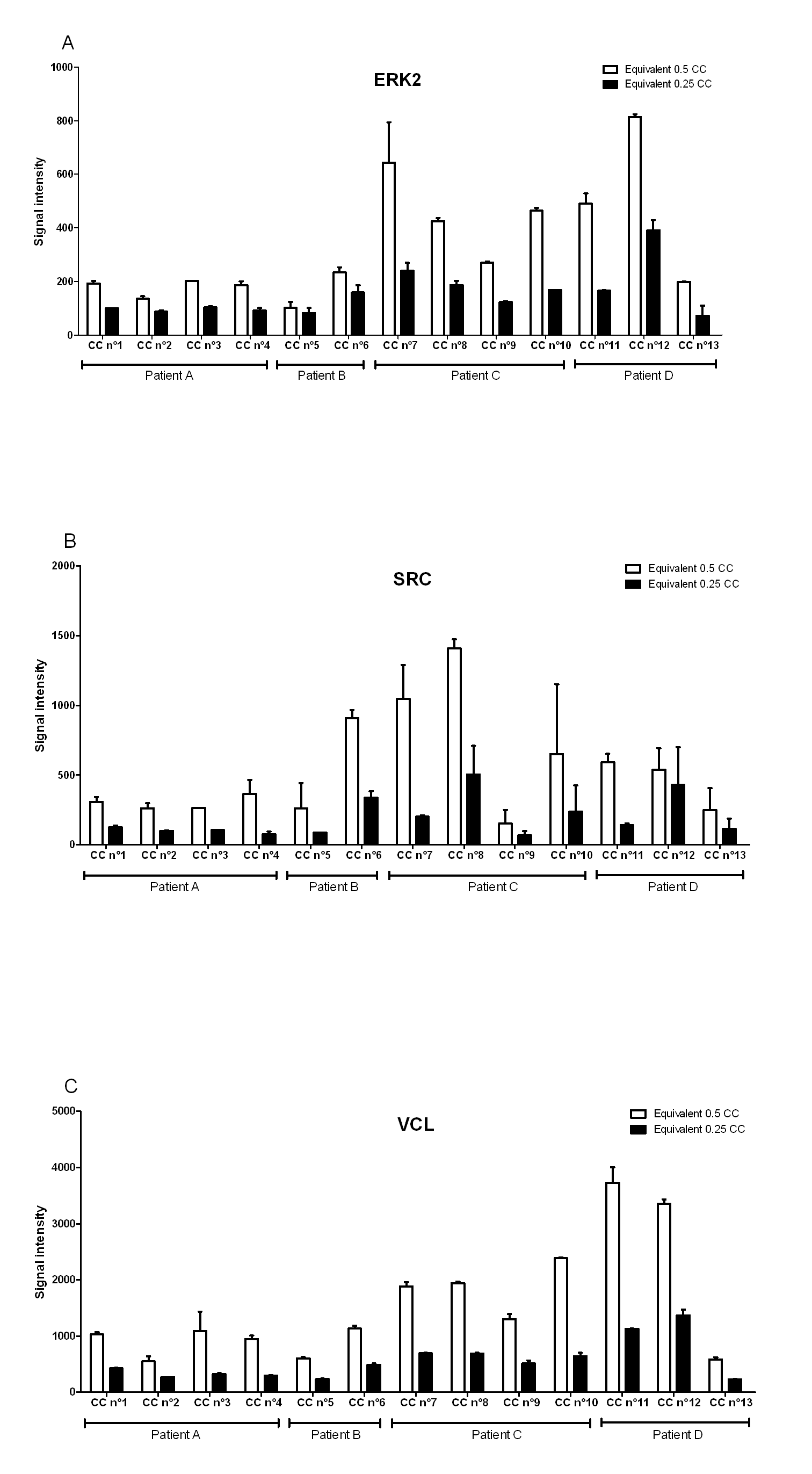

Supplement: Additional file 4: Figure S1 — Detection of ERK2, SRC and VCL proteins by Reverse Phase Protein Array on 13 individual cumulus cells. Detection of ERK2 (A), SRC (B) and VCL (C), by Reverse Phase Protein Array on 13 individual cumulus cells from four patients (A to D). The equivalent of 0.5 (white box) and 0.25 (black box) individual cumulus cells were spotted in two replicates on the array. Signal intensities are expressed as mean ± SD of the two replicates. [file 1477-7827-11-100-S4.tiff]
